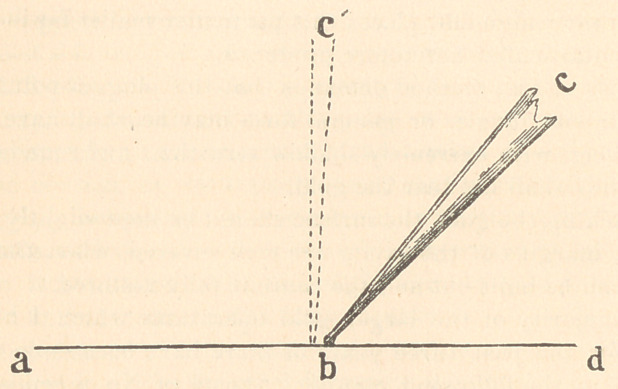# The Bonwill Method of Packing Gold Foil

**Published:** 1889-09

**Authors:** Edward C. Kirk

**Affiliations:** Philadelphia


### THE BONWILL METHOD OF PACKING GOLD FOIL.[1]

BY EDWARD C. KIRK, D.D.S., PHILADELPHIA.

THE method which I shall endeavor to describe to you is dependent on the use and the proper understanding of an automatic power-mallet delivering blows with great rapidity, and is possible only with an instrument of that character.

By automatic power-mallet I mean a mallet by which the blows are given in regular sequence with uniform rapidity and intensity,

---

[1] Read at the twenty-first annual meeting of the Pennsylvania State Society, held at Cresson, July 30, 1889.

and by means of power other than manual. Of such instruments we have two which fill the conditions I have prescribed: they are the Electro-magnetic and Engine Mechanical Mallets of Bonwill. The various other forms of engine-mallets which are on the market, so far as my experience with them goes, fail in two qualities essential in a mallet for packing gold by the Bonwill method,—viz., in rapidity and in quality of blow.

The idea which I wish to convey will probably be better understood if we examine for a moment the various stages of development which have taken place in the mallet for packing gold in teeth. We have the simplest expression in the hand-mallet, which has been modified indefinitely in form, size, weight, and material, to suit the fancy of individual operators, until the variety is almost infinite, but they all have the common characteristic of a uniform principle in their method of use, which is, briefly: When the gold has been introduced, and partially adapted or conformed to the cavity-walls, it is condensed, or rendered homogeneous, by holding a suitable instrument-point in contact with the gold, and delivering with the hand-mallet a blow upon the head of the instrument in contact with the gold; the force of the blow is conveyed through the steel shank of the plugging-instrument to the point, and expended upon the gold immediately under the point; the laminæ of foil are forced into close contact, or, technically, are "condensed." The point of the plugging-instrument is then moved to another portion of the gold surface, and the blow with the mallet repeated as before. The operation is continued until the whole mass of gold is introduced and made homogeneous in the same manner.

The tediousness of this method, and the time consumed in large operations, even under the most favorable circumstances, when the malleting was performed by a trained assistant, led to the introduction of that class of hand automatic mallets of which the Snow and Lewis automatic is probably the best and most widely known example. By the use of this mallet was gained extra facility in packing gold, as it combined both plugging-point and mallet in one instrument, thus allowing the operation to be done with the use of but one hand of the operator, and dispensing with the need for an assistant for malleting, with, in many cases, though not in all, a gain in speed.

There was, however, no change in principle, as regards the method of condensing the gold, from that pursued in the use of the hand-mallet,—viz., uniformly *dotting* the surface with blows from the mallet through the plugger.

The continued demand for a condensing instrument which would lessen the drudgery of gold filling operations, and the time necessary for their performance, brought into existence the electro-magnetic mallet of Bonwill, which involved an entirely new principle,—viz., the use of a power which rendered the instrument perfectly automatic, and increased the rapidity of the blows to an extent before unattained. The instrument was accepted by the profession, and used with the same general idea in packing gold as was customary, and, for that matter, necessary, in the use of all mallets previously used. In fact, Dr. Webb taught that the electrical mallet was to be held in the hand and applied to the gold in a manner similar to that of a pen in making dots on paper,—the principle being, the hand-mallet and plugger made absolutely automatic, and the blows repeated with infinite rapidity.

The engine-mallet of Bonwill not only embraced all that was of value in his electrical-mallet, but developed in his hands a method for the condensation of gold foil, and its adaptation to cavity-walls, which, in my judgment, places this particular mallet far in advance of any dental mallet heretofore produced.

The use of this method demands that the plugger-points, whatever their size, angle, or general form may be, shall have slightly convex faces, with extremely shallow serrations and rounded edges, so that they shall not tear the gold.

In packing the gold, the surface should be kept slightly concave until the margins of the cavity are well covered, when the central portion can be built out and the contour fully restored.

The majority of the larger gold operations which I have performed for the past three years or more have been done with foil prepared in the following manner: Sheets of No. 4 semi-cohesive "globe" foil are cut in half, and these halves again cut across, making four squares of foil. These are then crimped between foil crimpers, yielding a tape two inches long and one-eighth of an inch wide, and annealed. If the cavity is large and accessible, they are used full length; if small, they are cut to the desired length. One end of the tape is tacked into the cavity, and with the mallet it is woven in and malleted to place by the planishing or burnishing movement of the plugger from side to side, before described. The gold tapes made with the crimpers are used to fill the main body of the cavity, while for the restoration of contour, rolled "globe" foil No. 20 or 30 is used.

The use of foil in the manner described has enabled me to obtain satisfactory results with the least labor, and with a minimum expenditure of time. Dr. Bonwill, I believe, prefers to use exclusively the Abbey foil No. 20, rolled. The choice of foil will no doubt remain a matter of individual taste.

In the use of any hand-mallet the plugger-point is necessarily held at a right angle to the surface of the gold when the blow is delivered upon it, and during the delivery of the blow the point must be held in contact, or pushed against the gold, making it impossible to move or slide the instrument during the action of the blow, with the object of obtaining a lateral pressure on the gold surface. With the automatic power-mallets of Bonwill, the plugger-point should be held lightly in contact with the gold surface, *but at an oblique angle*, the effect being, when a properly-made point is used, to cause it to slip over the gold surface as the blows are delivered. I can, perhaps, illustrate this point, which is the main factor in the method of packing gold under consideration, better by means of a diagram.

*A d* represent the gold surface ; *b c* the plugger. When an impulsive force is applied at *c*, with the plugger in a position at a right angle to *a d*, the effect of the blow is expended upon the gold immediately beneath the point *b*, the tendency of the instrument to advance being overcome by the resistance of the gold surface.

With the plugger in an oblique position, an impulsive force applied at *c* not only effects a condensation of the gold immediately under the point *b*, but causes it to advance towards *a*, the result being that the foil is rubbed into position in much the same manner that it would be by a burnisher; with the exception that the force back of the instrument, instead of being continuous and uniform, is the interrupted, impulsive, percussive force of the mallet, and the point, instead of being polished like a burnisher, is slightly serrated.

The method which I have outlined, in my judgment, has several points of excellence, which not only make it worthy of special investigation, but place it far in advance of any method heretofore known for the introduction of gold.

The gold is brought into perfect coaptation with the cavity-walls, and is homogeneously condensed.

The operation can be done in much less time than by any other method that will do it as well.

The gold can be carried safely against and over the frailest walls of enamel, without fracturing them. To quote Dr. Bonwill, "The gold can be carried up the frailest walls, each piece reinforcing itself, and laying a floor up to the margins, giving a security that heavy blows from a mallet cannot. It enables one so to weave the foil by degrees over the edge or periphery of a cavity that there is always a fold of gold under the condensing tool."

The use of the mallet as described renders the homogeneous condensation of the foil a certainty. A practical study of the effect of the Bonwill engine-mallet in packing gold by this method will, I think, demonstrate to any dental practitioner, what has long seemed clear to my mind, that it embraces the best features of the condensing force of the Herbst method, combined with the greater efficiency of the mallet.

I have confined my remarks to a single feature of the use of the mallet, as practised and advanced by Dr. Bonwill, in the hope that the idea may help some of my professional brethren to reduce, in a measure, the physical and mental strain incident to the insertion of extensive gold fillings, and to make such operations a pleasure, instead of drudgery.

Much might yet be said upon the especial features of construction and management of the engine-mallet, but they do not fall within the prescribed limits of the subject of this paper.

## Figures and Tables

**Figure f1:**